# Spontaneous pneumomediastinum, pneumothorax and subcutaneous emphysema in critically ill COVID-19 patients: A systematic review

**DOI:** 10.12669/pjms.38.3.5529

**Published:** 2022

**Authors:** Kavous Shahsavarinia, Golnarz Rahvar, Hassan Soleimanpour, Mohammad Saadati, Leila Vahedi, Ata Mahmoodpoor

**Affiliations:** 1 Kavous Shahsavarinia Emergency Medicine Research Team, Road Traffic Injury Research Center, Tabriz University of Medical Sciences, Tabriz, Iran; 2 Golnaz Rahvar Emergency Medicine Research Team, Tabriz University of Medical Sciences, Tabriz, Iran; 3 Hassan Soleimanpour Emergency Medicine Research Team, Tabriz University of Medical Sciences, Tabriz, Iran; 4 Mohammad Saadati Khoy University of Medical Sciences, Khoy, Iran; 5 Leila Vahedi, MD-PhD Assistant Professor, Department of Medical Genetics, Liver and Gastrointestinal Diseases Research Center, Tabriz University of Medical Sciences, Tabriz, Iran; 6 Ata Mahmoodpoor, Anesthesiology Department, Faculty of Medicine, Tabriz University of Medical Sciences, Tabriz, Iran

**Keywords:** Pneumomediastinum, Pnemothorax, Emphysema, COVID-19

## Abstract

**Objectives:**

COVID-19 patients develop Life-threatening complications like pneumomediastinum/pneumothorax and emphysema which might experience prolonged hospital stays and additional costs might be imposed on the patient and the health system. The clinical features and outcomes of mechanically ventilated patients with COVID-19 infection who develop a pneumothorax, pneumomediastinum and subcutaneous emphysema has not been rigorously described or compared to those who do not develop these complications. So a systematic review of studies conducted on this subject was carried out to better manage these complications by investigating the underlying factors in COVID-19 patients.

**Methods:**

The search was conducted between early January and late December 2020 in databases including PubMed, Scopus, ProQuest, Embase, Cochrane Library, and Web of Science, using the following keywords and their combinations: COVID-19 Complication, Pneumothorax, Pneumomediastinum, Pneumopericardium, and Subcutaneous Emphysema. The extracted studies were screened separately by two researchers based on the PRISMA statement. After eliminating the duplicate studies, the title, abstract, and full text of the remaining studies were reviewed. Disagreements in the screening and selection of the studies were resolved by consensus or through a third-party opinion.

**Results:**

A total of 793 articles were retrieved through the literature search, and 99 studies conducted on a total of 139 patients were finally included The patient mortality was found to have a significant relationship with positive pressure ventilation (P=0.0001). There was no significant relationship between the patients’ death and chest tube insertion (P=0.2) or between the interval of time from the onset of symptoms to the diagnosis of pneumothorax (P=0.7). The mean age was higher in the deceased cases, and the mean difference observed was statistically significant (P=0.001).

**Conclusion:**

With the expansion of our clinical understanding of COVID-19, recognition of the uncommon complications of COVID-19 especially pneumothorax is crucial. Although in our review we couldn’t find a causal relationship between COVID-19 and pneumothorax or association between pneumothorax and death, as it is limited by many variables such as included studies’ design, or incomplete outcome data especially more information about the associated risk factors, we recommend performing more well-designed studies to describe the pneumothoraxes’ incidence, risk factors, and outcomes in COVID-19 patients.

## INTRODUCTION

COVID-19 is an infectious disease caused by the coronavirus. This disease has been spreading across most countries since November 2020 and the COVID-19 pandemic has turned into a serious global health threat.^1^-^4^ Although most COVID-19 patients exhibit mild symptoms and have a good prognosis, this disease may lead to life-threatening conditions.^5^-^8^ Pneumothorax, tension pneumothorax, pneumomediastinum, pneumopericardium and subcutaneous emphysema are uncommon clinical manifestations in patients with COVID-19. These patients experience spontaneous pneumothorax more commonly (0.57%) than other critical patients.^1^ The odds of developing spontaneous pneumothorax is 40 to 100 times higher in patients with COVID-19 compared to those without, and mechanical ventilation increases the risk of pneumothorax in COVID-19 patients.^5^,^6^ According to a study, the pathophysiology of pneumomediastinum in COVID-19 patients can be explained by the Macklin effect.^7^ The Macklin effect starts with alveolar rupture secondary to direct alveolar injury, leading to air leaking and dissection along the bronchovascular sheaths and eventually spreading of air within the mediastinum. This leaking of air can be aggravated by intense coughing leading to a sudden increase in distal airway pressure and ultimately alveolar rupture. Despite using a low tidal volume during ventilation, there was no relationship between pneumomediastinum/subcutaneous emphysema and the classic barotrauma mechanism; therefore, when barotrauma is eliminated as the cause, the underlying disease that is, COVID-19 appears to be the cause of these manifestations through the Macklin effect and lung frailty.^8^

The clinical features and outcomes of mechanically ventilated patients with COVID-19 infection who develop a pneumothorax, pneumomediastinum and subcutaneous emphysema has not been rigorously described or compared to those who do not develop these complications. Given that these conditions are life-threatening and COVID-19 patients developing such complications might experience prolonged hospital stays and additional costs might be imposed on the patient and the health system, a systematic review of studies conducted on this subject was carried out to better manage these complications by investigating the underlying factors causing pneumothorax, pneumomediastinum, and subcutaneous emphysema in COVID-19 patients.

## METHODS

### Search strategy and selection criteria

The search was conducted between early January and late February 2021 in databases including PubMed, Scopus, ProQuest, Embase, Cochrane Library, and Web of Science, using the following keywords and their combinations: COVID-19 Complication, Pneumothorax, Pneumomediastinum, Pneumopericardium, and Subcutaneous Emphysema.

### Inclusion criteria

Articles that met the following criteria were included in our study: 1) studies that described pneumothorax on chest imaging [using either chest radiography or chest computed tomography (CT)] in hospitalized adults and children due to COVID-19 infections; 2) observational studies, including cohort, case-control, and cross-sectional studies; 3) studies written in English language; 4) studies in which diagnosis of COVID-19 infections was made via real-time reverse transcription-polymerase chain reaction (RT-PCR) from nasopharyngeal or oropharyngeal swab; 5) published between January 1st, 2020 to Feb 30th, 2021 in a peer-review journal; and 6) studies addressing at least one of the following issues: a) incidence, b) risk factors, c) onset, and/or d) outcome of COVID-19-related pneumothorax.

### Exclusion criteria

The exclusion criteria were specified as follows: 1) studies that only reported signs of barotraumas such as pneumomediastinum and subcutaneous emphysema in the absence of pneumothorax in hospitalized COVID-19 patients; and 2) studies describing iatrogenic causes (e.g., from central venous catheter insertion) of pneumothorax in COVID-19 patients.

### Screening, selection and quality assessment

The extracted studies were screened separately by two researchers based on the PRISMA statement. After eliminating the duplicate studies, the title, abstract, and full text of the remaining studies were reviewed. Disagreements in the screening and selection of the studies were resolved by consensus or through a third party opinion. This process was managed using Endnote-8. All case report or case series studies conducted on COVID-19 patients with pneumothorax, pneumomediastinum, pneumopericardium, or subcutaneous emphysema were included; however, the case series studies reporting the cases collectively were excluded. The quality assessment of the articles was performed by one of the researchers and their random reassessment was done by another researcher. The JBI Critical Appraisal Checklist for Case Reports was used to assess the quality of the case reports.

### Data extraction

Data were extracted from the studies using an Extraction Table designed according to the objectives by one of the researchers and through random assessment by another researcher. The extracted data included patient’s age and gender, presence or absence of pneumothorax, pneumomediastinum, pneumopericardium, tension pneumothorax , pulmonary involvement, recurrent pneumothorax, induced disease post PAP or intubation, early signs, danger signs, history of disease, SPO_2_ at admission, time of onset of symptoms and pneumothorax diagnosis, means of diagnosis of pneumothorax, chest tube insertion or non-insertion, medication measures, duration of hospitalization, and patient outcomes (deceased or survived). The reported signs are related to both COVID-19 and pneumonthorax/penumomediastinum or subcutaneous emphysema.

### Data synthesis and analysis

The extracted data were summarized and analyzed in Excel using descriptive statistics, including mean and standard deviation and frequency and percentage frequency. The inferential analysis and assessment of the relationship between the variables were carried out in SPSS-20.

## RESULTS

**Fig.1:**
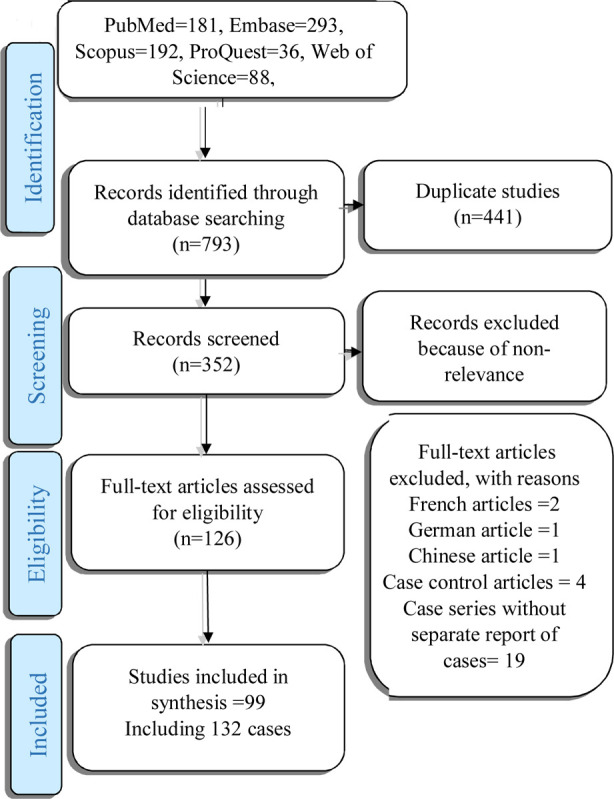
PRISMA flow diagram of the study.

A total of 793 articles were retrieved through the literature search, and 99 studies conducted on a total of 139 patients were finally included (Fig.1). The patients’ mean age was 55.19±16.1 years, and the majority were men (83.1%). According to the results, 96.8% of the patients had pulmonary involvement at the time of admission to the hospital, and 67.4% suffered from pneumothorax following infection with COVID-19. Pneumothorax and pneumomediastinum had no significant relationship with the patients’ age or gender (P>0.05).

**Table I:** Characteristic of patients.

*Average Age*	*Gender = M/F*	*Percentage frequency of if the patient had pulmonary involvement at admission*	*Percentage frequency of At which admission did the PT, PM…occur?*	*Percentage frequency of Recurrent Pneumothorax*	*Percentage frequency of Did PT, PM,… occur after intubation or PAP ventilation?*	*Past Medical History*	*The interval between admission and PT occurrence*	*PT, PM, … diagnose method*	*Chest tube insertion*	*Intubation*	*Mechanical ventilation*	*Hospitalization duration (mean)*	*Mortality*	*Recovery*

55.18	5.1	96.8	First time (86.7%), second time (13.3%)	9.9	28.8	43.9	15.2	CT scan (54.3%), X-ray (34.9%). X-ray then CT (10.9%)	51.5	27.3	39.2	24.5	24.8	75.2

According to the examined case reports, 28.8% of the patients had developed pneumothorax following intubation or CPAP. The most common symptoms included shortness of breath (70.3%), fever (61.7%), and dry cough (58.6%). The review of the case reports revealed that 89.8% of the patients had at least one pulmonary symptom. Pneumothorax was diagnosed at the time of admission in 33.9% of the patients, and the mean duration from the onset of symptoms to the diagnosis of pneumothorax was 15.21±10.2 days. The most common method for diagnosing pneumothorax was CT scan (54.3%), followed by X-ray (34.9%). In other cases, both methods had been used. Chest tubes had been inserted for 51.5% of the patients and 27.3% were intubated. A total of 18.9% of the COVID-19 patients with pneumothorax, 14.4% of those with pneumomediastinum, 12.9% of those with emphysema, 6.1% of those with hemothorax and 1.5% of those with pneumopericardium had been intubated. There were no reports on the risk factors of COPD (bronchitis, emphysema, and smoking) in over half of the included studies (53%). In the remaining cases, 74.2% of the patients had no risk factor, 24.2% were smokers, and 1.6% had emphysema. Using marijuana with cigarettes was reported as a risk factor in one of the patients. In addition to risk factors, previous history of diseases was a noteworthy item, but it was not reported in 56.1% of the studies. Hypertension (50%), diabetes (41.3%) and obesity (17.2%) were reported as the most common previous diseases in the patients.

NIPPV had been used in 39.2% of the patients. This rate was 24.6% in the patients with pneumothorax. Pneumothorax was not found to have a significant relationship with positive pressure ventilation in the patients (P>0.05). The mean duration of hospital stay was 24.5 days, and one of the patients died two hours after admission. Investigating the patients’ outcome revealed that 24.8% of the reported cases were deceased. The patient mortality outcome was found to have a significant relationship with positive pressure ventilation (P=0.0001). There was no significant relationship between the patients’ death and chest tube insertion (P=0.2) or between the interval of time from the onset of symptoms to the diagnosis of pneumothorax (P=0.7). The mean age was higher in the deceased cases, and the mean difference observed was statistically significant (P=0.001). The relationship between the mean SPO2 at the time of admission and death was close to significant (P=0.056).

## DISCUSSION

According to the results of previous studies, pneumothorax was more prevalent in men (ratio of 3.3:1),^9^ which confirms that men are more affected by severe forms of the disease.^10^ Although most patients in the present analysis were middle-aged men, the results showed that pneumothorax and pneumomediastinum have no significant relationship with the patients’ age and gender. A case-control study conducted by Miro et al. showed no gender difference between the occurrence of pneumothorax (72.5% vs. 51.3%; P>0.05).^2^

**Table II:** Side effects frequency in included cases.

Title	Number (%)
Pneumothorax (PT)	89 (67.4)
Pneumomediastinum (PM)	70 (53)
Pneumopericardium (PP)	32 (24.2)
Subcutaneous Emphysema (SE)	30 (22.7)
Tension Pneumothorax (TP)	29 (22)
Hemothorax (HTX)	8 (6.1)
Parallel PM & PT	31 (23.5)
Parallel TP & PT	28 (21)
Parallel HTX & PT	7 (5.3)

The pathophysiology behind spontaneous lung injury is still under investigation, and can be due to several theories including direct viral damage, microthrombosis, exaggerated immune response and obesity-related.

The results of the previous studies showed that pneumothorax had been diagnosed in over one-third of the patients at the time of admission, and the mean interval between the onset of symptoms and the diagnosis of pneumothorax was 15.21±10.2 days.^11^-^13^ Chong et al. reported the incidence of pneumothorax (about 0.3%) in hospitalized patients with COVID-19 and between 12.8% and 23.8% in patients requiring mechanical ventilation.^14^

Cyst formation in areas of airspace disease was first considered immediately after initial onset as a COVID-19 radiological finding and was confirmed by studies showing radiological progression from consolidation bullae to bullae regions.^15^-^17^ Previous reported cases have shown that cyst formation is not merely limited to patients under positive pressure ventilation, which shows that barotrauma alone cannot explain these findings.^18^ The fact that many pneumothorax cases are reported in patients who have not been under mechanical ventilation confirms that this relationship cannot be explained by barotrauma alone. Cyst formation has also been cited as a delayed outcome of SARS-induced ARDS, assumed processes of the disease, including ischemic parenchymal damage, and inflammation.^19^ In terms of intensive care admissions, previous analyses of intubated SARS patients showed that tachypnea at the time of admission, hypoxemia, and hypercapnia are all associated with the progression of pneumothorax, but ventilator pressure or volume variables did not have a significant effect.^20^ Aiodfi et al. reported two cases of COVID-19 patients who developed persistent pneumothorax under mechanical ventilation.^20^ Meanwhile, there are somereports who developed pneumothorax, pneumomediastinum, and subcutaneous emphysema without any mechanical ventilation.^19^,^21^

Gattinoni et al. found that the incidence of pneumothorax was higher in patients with ARDS who had been under prolonged mechanical ventilation (87% vs. 30% in those who had been mechanically ventilated for longer than two weeks compared to those less than one week). In addition, a higher rate of pneumothorax was reported in the patients with bullae and low lung compliance.^22^ Despite its relative rareness, spontaneous pneumomediastinum has been reported in association with ARDS related to coronavirus pneumonia in some cases^23^ and has been connected to a high incidence of other viral pneumonias in some cases, including H1N1 influenza. Although its exact mechanism is unknown, increased alveolar pressure and diffuse alveolar damage are common in severe COVID-19 pneumonia, which is likely to predispose the alveoli to rupture, especially in patients with a distinct cough.^24^,^25^

The pathophysiology of increased incidence of pneumomediastinum in COVID-19 patients is not clear. In a retrospective study conducted by McGuinness G et al., higher rates of barotrauma were observed in COVID-19 patients under invasive mechanical ventilation than in patients with ARDS and those not infected with COVID-19.^26^ COVID-19-induced structural changes in the lungs and lung damage associated with mechanical ventilation may be responsible for pneumomediastinum in these patients. Spontaneous pneumomediastinum in COVID-19 patients may be caused by diffuse alveolar injury due to cytokine storm or direct viral infection of type I and II pneumocytes, making the alveoli more exposed to membrane rupture, resulting in air escaping from blood circulation through the sheaths surrounding the bronchi and perivascular to the mediastinum, known as the “Macklin phenomenon”.^27^,^28^

The early diagnosis of this complication is highly important, since one episode of pneumothorax can be a risk factor for recurrent pneumothorax.^29^ Although the actual incidence of pneumothorax in COVID-19 patients under mechanical ventilation is not yet clear, the set of cases reviewed in this study suggest that this number is significant, which should be borne in mind if there is a sudden change in the clinical status of an intubated patient. The ongoing use of ventilation strategies to protect the lungs and interventions to prevent secondary bacterial infections will also be helpful.^30^

The present systematic review is the largest study that analyzes the results from reports on cases of pneumothorax, pneumomediastinum, and subcutaneous emphysema cited in case report or case series studies on COVID-19 that include non-ventilated patients as well.

### Limitations

There were several limitations in the present study. There are often limited data on pneumothorax in COVID-19 infections due to the large frequency of variables reported in many observational studies compared to other common chest CT findings. This issue can be explained by the inconsistent use of chest imaging, as almost all the COVID-19-related pneumothoraces are diagnosed by chest CT scan in observational studies compared to chest radiography. A delay might occur in the diagnosis of pneumothorax, since chest CT scan is more sensitive than chest radiology in diagnosing pneumothorax. This issue highlights the importance of normal chest X-ray in ensuring the quick detection of this unusual matter in COVID-19 infections. Some patients with mild symptoms of COVID-19 who are not diagnosed with pneumothorax may be advised to stay at home during the outbreak to prevent further transmission of COVID-19 by hospitalization.

## CONCLUSION

With expansion of our clinical understanding of COVID-19, recognition the uncommon complications of COVID-19 especially pneumothorax is crucial. Although, in our review we couldn’t find a causal relationship between COVID-19 and pneumothorax or association between pneumothorax and death, as it is limited by many variables such as included studies’ design, or incomplete outcome data especially more information about the associated risk factors, we recommend to perform more well-designed studies to describe the pneumothoraxes’ incidence, risk factors, and outcomes in COVID-19 patients.

### Authors’ Contribution:

**KS:** Study hypothesis, literature review, collecting data, initial drafting. Responsible and accountable for the accuracy or integrity of the work. **GR:** Study design, initial drafting, manuscript editing. **HS:** Literature review, collecting data, manuscript editing. **MS:** Study hypothesis, literature review, initial drafting. **LV:** Study design, initial drafting, manuscript editing. **AM:** Collecting data, literature review, Data analysis. Responsible and accountable for the accuracy or integrity of the work. All authors read the final edition and approved the final edition of the manuscript.
